# A Molecular, Genetic, and Diagnostic Spotlight on Fanconi Anemia

**DOI:** 10.1155/2012/650730

**Published:** 2012-10-02

**Authors:** Laura E. Hays, Stefan Meyer, Henri J. van de Vrugt

**Affiliations:** ^1^Division of Hematology & Oncology, School of Medicine, Oregon Health & Science University, 3710 SW US Veterans Hospital Road, R&D-2, Portland, OR 97239, USA; ^2^Stem Cell and Leukaemia Proteomics Laboratory and Paediatric and Adolescent Oncology, University of Manchester, Royal Manchester Children's Hospital and The Christie NHS Foundation Trust, Manchester Academic Health Science Centre, Manchester M20 4BX, UK; ^3^Division of Biological Stress Response, The Netherlands Cancer Institute, Plesmanlaan 121, 1066 CX Amsterdam, The Netherlands

Eighty-five years ago Dr. Guido Fanconi described a family with three brothers with microcephaly, hyperpigmentation of the skin, hypoplasia of the testes, and who developed a lethal anemia between the ages of 5 and 7 [1, 2]. Since 1931, patients with this distinctive combination of clinical features have been classified as having Fanconi anemia (FA). To date, diagnosis of this disease, which as we know can manifest with variable clinical presentation, is based on increased chromosomal breakage in response to DNA crosslinking agents.

In addition to anemia, FA patients are highly predisposed to leukemia, head and neck squamous cell carcinoma, and gynecological cancers [3]. FA is caused by biallelic or X chromosome-linked mutations in one of 15 different genes, eight of whose gene products (FANCA, FANCB, FANCC, FANCE, FANCF, FANCG, FANCL, and FANCM) form a nuclear core complex that must be intact to ubiquitinate FANCD2 and FANCI. The ubiquinated FANCD2/FANCI heterodimer then functionally interacts with down-stream FA proteins (FANCD1/BRCA2, FANCJ/BRIP1, FANCN/PALB2, FANCO/RAD51C, and FANCP/SLX4) to mediate DNA damage responses [4].

This special issue provides a wide range of scientific papers and reviews that examine in detail techniques for diagnosis of FA, molecular and genetic mechanisms of FA protein function, comprehensive details of the varied clinical manifestations, new model systems for study of this disease, and potential new therapeutics. Three papers describe FA relevant aspects in FA diagnoses using chromosomal breakage analysis and mutation detection by multiplex ligation-dependent probe amplification (MLPA) and PCR-based Sanger sequencing, or next generation sequencing. The strategy to detect mutations and the genetic counseling of involved families should be adapted when there is evidence for a founder effect, which results in a high prevalence of a common mutation in a specific population. In this special issue, Y. de Vries et al. provide evidence that FA-C patients with the *FANCC *c.67delG mutation in the Dutch and Canadian Manitoba Mennonite populations originate from a common founder.

One impediment to FA research has been the relative lack of a murine model that truly recapitulates the severity of FA hematopoietic defects. In the data reported by J. H. E. Verhagen-Oldenampsen et al., Ercc1-deficient mice were used as model for FA-like bone marrow failure. Although no *ERCC1* mutations have been detected in FA patients, ERCC1 interacts with FANCP/SLX4 and functions in interstrand crosslink repair [5], the critical type of DNA damage recognized and repaired by the FA/BRCA pathway [6] (Figure 1).

Another group of papers in this issue provides detailed and thorough scientific reviews of the varied clinical and molecular aspects of this disease, new model systems, and potential future treatments. First, the review by M. D. Milsom et al. details FA-associated defects in hematopoietic stem cell biology and resultant bone marrow failure. FA chromosomal aberrations associated with clonal evolution and leukemic transformation are the focus of the review by S. Meyer et al. T. Kaddar and M. Carreau review aspects of FA protein function that have been placed in the shadow by the recent focus on the role of the FA pathway in DNA repair. The paper by C. Hodson and H. Walden comprehensively reviews aspects of protein interactions and function in the FA core complex, while the review by M.R. Jones and A.M. Rose focuses on utilization of a relatively new model system for FA, the worm *C. elegans. *They examine the functions of DOG1, a functional ortholog of FANCJ, and FANCD2 in interstrand crosslink repair. To conclude the special issue, Jenkins et al. summarize the efforts that have been made to generate and utilize FA pathway inhibitors as novel anticancer therapies.

The great progress in understanding FA on a molecular, cellular and clinical level illustrated in this special issue has made a big difference to people affected by FA, but also an enormous contribution to hematology, cancer, and developmental biology research. This has been achieved to a large extent through close interactions between scientists, clinicians and patients, which provide a resourceful platform for not only exchange and stimulation, but also a constant reminder of the goals of this research—aiming to understand biology in order to make the journey of families with FA a more hopeful one.

**FA Patient Support Groups by Bob Dalgleish, Fanconi Hope, UK**

FA patients, although relatively few in number in each country, are fortunate in having a number of strong family support groups that coordinate their activities on an international basis to ensure a coherent approach to supporting research. The longest established group, the United States-based Fanconi Anemia Research Fund (FARF: http://www.fanconi.org.uk/) has been responsible for sponsoring a significant amount of research for over 20 years. Also in existence over a similar period, the German group Deutsche Fanconi-Anämie-Hilfe e.V. (Fanconi Anemia Aid Association: http://www.fanconi.de/), has been actively involved on an international scale in long-term research programs. The more recently formed UK-based organization, Fanconi Hope (http://www.fanconi.org.uk/) has been supporting research through the auspices of the FARF Scientific Advisory Board, for UK research projects selected by FARF.

Fanconi Hope and FARF also sponsor an International FA Gene Therapy Working Group, now in its third year, which aims to accelerate the transition from research to gene therapy trials for FA patients by coordinating activities on an international scale. FA patients, their families and friends are in large part responsible for raising the funds for this research. This is of great benefit to those affected as this allows them to believe they can make a difference, if not to the current generation of FA patients then at least as a legacy to the benefit of the next generation.

*Laura E. Hays* *Stefan Meyer* *Henri J. van de Vrugt*

## Figures and Tables

**Figure 1:**
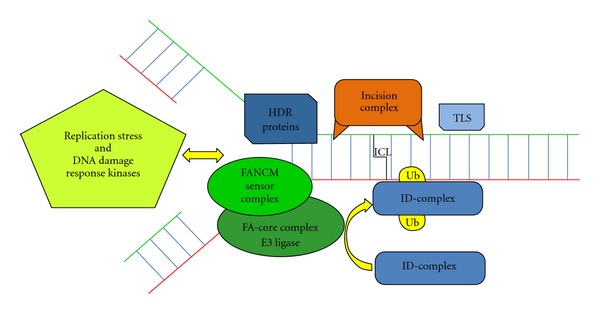
Schematic overview of the FA pathway. The FA pathway operates to maintain genomic stability in response to stalled replication forks, particularly in the context of interstrand DNA crosslinks (ICLs) that covalently link the two strands of the DNA helix. Replication stalling is detected by the FANCM sensor complex and triggers the activation of the Rad3-related protein kinase ATR, resulting in the phosphorylation of several FA proteins. FANCM becomes a docking platform for the other FA core complex proteins including the FANCL E3 ubiquitin ligase. The core complex activates the two members of the ID complex, FANCD2 and FANCI, by monoubiquitination (Ub) to promote ICL repair. The incision complex includes FANCP/SLX and ERCC1 and is essential to resolve the crosslink. Repair and replication fork restart is further accomplished using translesion synthesis (TLS) and homology-directed repair (HDR) which involves BRCA2/FANCD1, PALB2/FANCN, and RAD51C/FANCO.
